# Efficient therapy of ischaemic lesions with VEGF_121_-fibrin in an animal model of systemic sclerosis

**DOI:** 10.1136/annrheumdis-2015-207548

**Published:** 2015-09-11

**Authors:** Shadab Allipour Birgani, Marion Mailänder, Ines Wasle, Hermann Dietrich, Johann Gruber, Oliver Distler, Roswitha Sgonc

**Affiliations:** 1 Division of Experimental Pathophysiology and Immunology, Biocenter, Medical University of Innsbruck, Innsbruck, Austria; 2 Central Laboratory Animal Facilities, Medical University of Innsbruck, Innsbruck, Austria; 3 Department of Internal Medicine VI, Medical University of Innsbruck, Innsbruck, Austria; 4 Department of Rheumatology, Center of Experimental Rheumatology, University Hospital Zurich, Zurich, Switzerland

**Keywords:** Systemic Sclerosis, Treatment, Outcomes research

## Abstract

**Background:**

In systemic sclerosis (SSc), chronic and uncontrolled overexpression of vascular endothelial growth factor (VEGF) results in chaotic vessels, and intractable fingertip ulcers. Vice versa, VEGF is a potent mediator of angiogenesis if temporally and spatially controlled. We have addressed this therapeutic dilemma in SSc by a novel approach using a VEGF_121_ variant that covalently binds to fibrin and gets released on demand by cellular enzymatic activity. Using University of California at Davis (UCD)-206 chickens, we tested the hypothesis that cell-demanded release of fibrin-bound VEGF_121_ leads to the formation of stable blood vessels, and clinical improvement of ischaemic lesions.

**Methods:**

Ninety-one early and late ischaemic comb and neck skin lesions of UCD-206 chickens were treated locally with VEGF_121_-fibrin, fibrin alone, or left untreated. After 1 week of treatment the clinical outcome was assessed. Angiogenesis was studied by immunofluorescence staining of vascular markers quantitatively analysed using TissueQuest.

**Results:**

Overall, 79.3% of the lesions treated with VEGF_121_-fibrin showed clinical improvement, whereas 71.0% of fibrin treated controls, and 93.1% of untreated lesions deteriorated. This was accompanied by significantly increased growth of stable microvessels, upregulation of the proangiogenic VEGFR-2 and its regulator TAL-1, and increase of endogenous endothelial VEGF expression.

**Conclusions:**

Our findings in the avian model of SSc suggest that cell-demanded release of VEGF_121_ from fibrin matrix induces controlled angiogenesis by differential regulation of VEGFR-1 and VEGFR-2 expression, shifting the balance towards the proangiogenic VEGFR-2. The study shows the potential of covalently conjugated VEGF-fibrin matrices for the therapy of ischaemic lesions such as fingertip ulcers.

## Introduction

Systemic sclerosis (SSc, scleroderma), a devastating autoimmune connective tissue disease affecting the skin and viscera, is characterised by microangiopathy, immunological abnormalities and fibrosis.^1^ Although great progress has been made in unscrambling the complex pathogenic interactions between the immune system, the vasculature and fibrotic processes, the ultimate aetiology still remains unclear.^2^ However, apoptosis of endothelial cells (ECs) is considered to be a primary event in the pathogenesis of SSc that precedes inflammation and fibrosis.^3^

The search for the ultimate aetiology as well as the evaluation of new therapeutic approaches requires appropriate animal models. The University of California at Davis (UCD) chicken lines 200 and 206 are the only animal model displaying all hallmarks of SSc, that is, microvascular damage, severe perivascular mononuclear cell infiltration, and fibrosis of skin and viscera, circulating antinuclear antibodies, and anti-EC antibodies.^4^

Vascular alterations in human and avian SSc predominantly affect the microvasculature with intimal proliferation, occlusion of blood vessel and capillary necrosis, leading to a decreased blood flow, a state of chronic ischaemia and clinical manifestations such as fingertip ulcers and comb lesions.^5^ ^6^ Tissue hypoxia normally induces neoangiogenesis, but in SSc vascular repair and angiogenesis seem to be strongly disturbed.^7–9^ One of the key molecules in the induction of angiogenesis is vascular endothelial growth factor (VEGF). It is involved in several steps of angiogenesis including migration, proliferation and survival of ECs.^10^ In patients with SSc, VEGF expression is upregulated, but seems to be uncontrolled and chronic.^11–14^ Sufficient angiogenesis depends on the tight regulation of VEGF expression.^15^ Prolonged exposure to VEGF results in the formation of a chaotic capillary network with irregularly shaped, dilated capillaries, a morphology similar to that seen in SSc.^16^ ^17^ Thus, the chronic VEGF overexpression found in SSc might paradoxically lead to a disturbed vessel morphology rather than to promote the formation of new functional and stable vessels. Recently, increased serum levels of the antiangiogenic VEGF_165b_ isoform have been reported in patients with SSc, which could at least partly explain the lack of sufficient angiogenesis despite strongly elevated VEGF levels.^18^ However, very high levels of VEGF are associated with the absence of fingertip ulcers.^11^ ^19^ This suggests that the concentrations of proangiogenic VEGF isoforms have to exceed a certain threshold level to overcome the inhibitory effects of antiangiogenic factors, and that a further increase of VEGF might have beneficial effects in the prevention and therapy of fingertip ulcers. Yet, therapeutic VEGF can play either a helpful or harmful role in tissue vascularisation, depending on the dose and pharmacokinetics of its administration.^16^ ^20^ ^21^ Hence, uncontrolled long-term overexpression as in SSc or a burst release leads to chaotic morphology of the newly formed vessels with reduced blood flow, whereas a short-time upregulation of VEGF initiates angiogenesis, which results in the formation of stable blood vessels.^22^ Thus, in SSc, there is a therapeutic need for a temporally and spatially controlled availability of proangiogenic VEGF at sites of ischaemia (eg, fingertip ulcers).

In nature, the longer VEGF isoforms are bound to extracellular matrix (ECM) components until liberated in a tightly controlled manner by local enzymatic activity of cells invading the matrix.^23^ The ECM provides a bioactive dynamic structure, which controls EC activities by various mechanisms ranging from cell anchorage and growth factor binding to integrin-mediated activation, and thus is crucial for functional and sustained vascular growth.^24^ In order to mimic nature, Andreas Zisch *et al*^25^ have developed a method to covalently bind VEGF_121_ to a fibrin matrix, in which VEGF_121_ is released upon demand by enzymatic cleavage. Grafting experiments with this fibrin-bound VEGF variant demonstrated controlled growth of morphologically normal blood vessels.^26^ Based on this evidence, we hypothesised that local administration of VEGF_121_-fibrin and consequent cell demanded release of VEGF from the fibrin matrix should overcome the uncontrolled VEGF expression found in SSc, and induce sufficient angiogenesis to heal and prevent ischaemic ulcers. Here we present the therapeutic efficacy of this novel approach in the spontaneous avian model of SSc.

## Materials and methods

### Animals and study design

UCD-206 chickens were bred and maintained at the Central Laboratory Animal Facilities of the Medical University of Innsbruck. Animal breeding, housing, treatment and collection of tissue samples were carried out in accordance with the Animal Experiment Directive of the European Union (Directive 2010/63 EU) and the Austrian law on the protection of animals used for scientific purposes (‘Tierversuchsgesetz’ BGBL I Nr. 501/1989 idF 2005 and BGBL I Nr. 114/2012) after approval by the Federal Ministry of Science and Research (GZ 66.011/082-C/GT/2007, GZ 66.011/082-C/GT/2010, and GZ 66.011/077-WF/II/3b/2014). The clinical stage of early skin lesions is classified in the comb as C+ (erythema), C++ (erythema and oedema), C+++ (ulceration with onset of necrosis), C++++ (comb lost due to self dubbing) in the neck skin as N+ (erythema), N++ (erythema and oedema), and N+++ (ulceration with onset of necrosis), N++++ (fibrosis).^3^ Comb lesions developed between 9 days and 27 days of age (mean 14.7±4.1 SD), neck lesions between 22 days and 37 days of age (mean 26.0±3.9 SD).

For the present study, lesions were treated at the stages of C++ and C+++, neck lesions at stage N+++ either with VEGF_121_ modified fibrin gel, with fibrin gel only, or left untreated (comb lesions n=10 per group, neck lesions treated with VEGF_121_-fibrin or fibrin n=11 each, untreated n=9). Chickens were randomly assigned to receive VEGF_121_-fibrin, fibrin or no treatment, and received a code for blinded clinical and histological analyses.

### Procedures

In order to mimic the natural binding of VEGF to ECM, we used VEGF_121_ modified with the factor XIIIa substrate sequence NQEQVSPL. This variant, α_2_PI_1-8_-VEGF_121_, spontaneously cross-links to fibrinogen by the transglutamination activity of factor XIII (FXIII) during fibrin polymerisation.^25^ VEGF_121_-fibrin gel matrices were prepared by mixing 20 μg/mL α_2_PI_1-8_-VEGF_121_ (kindly provided by A Zisch) with fibrinogen solution (Tisseel, Baxter AG, Vienna, Austria) diluted with sterile Tris buffered saline (pH 7.2) to a final concentration of 10 mg/mL fibrinogen, and 2 U/mL FXIII (Baxter AG, Vienna, Austria) prior to initiation of fibrin gelation by addition of 2 U/mL thrombin (Tisseel, Baxter AG, Vienna, Austria). For placebo control, α_2_PI_1-8_-VEGF_121_ was omitted. The mixture was immediately applied to the lesion with the help of a small plastic ring to keep it in place till gel was formed, which took 20–30 s. The ring was then removed, and the gel covered with Opsite Flexigrid (Smith & Nephew, Hull, UK) to keep it moist.

After 7 days, the dressing was removed; pictures were taken for documentation and macroscopic analysis by comparison with pictures taken before treatment. The animals were sacrificed under Narkodorm anaesthesia (Pentobarbital, CP-Pharma, D-31303 Burgdorf, Germany, 20 mg/kg intravenous) by cardiac exsanguinisation. Combs and neck skin biopsies were shock frozen in liquid nitrogen and stored at −196°C till further analyses. The clinical outcome was evaluated by three experts (HD, JG and RS) independently, and in a blinded manner. The degree of change was scored as follows: 0=no change, +1=improvement, +2=strong improvement, −1=deterioration, −2=strong deterioration.

### Immunofluorescence staining

Angiogenesis was studied by indirect immunofluorescence (IIF) tests on 4 μm frozen tissue sections using antibodies specific for the EC marker von Willebrand factor (vWF), and α smooth muscle actin (αSMA) or desmin, which are expressed by pericytes (PCs) and smooth muscle cells (SMCs), and serve as markers for stable blood vessel formation. Expression of VEGF, VEGFR-1, VEGFR-2 and TAL-1 by ECs was also analysed by IIF double staining with the respective antibody and anti-vWF antibody. Nuclei were stained with DAPI. See online supplementary text for details on antibodies and staining procedure.

### Microscopy and quantitative analysis

IIF staining was analysed in a blinded manner using a Nikon Eclipse E800 fluorescence microscope (Nikon, Tokyo, Japan). For quantitative analysis digital black and white (b/w) pictures were acquired from 10 fields of view per tissue section using a 20× objective lens (Plan Apo 20×/0.75 DICM α/0.17 WD 1.0) with filter settings for FITC, RRX and DAPI in series (jpeg fine, size 1280×960, source s1.3M, CCD mode).

ECs and non-ECs expressing VEGF, VEGFRs or TAL-1 were quantified using the image analysis software TissueQuest cytometer (TQ 4.0 TissueQuest Software, TissueGnostics, Vienna, Austria). This microscopy-based multicolour tissue cytometry software permits multicolour analysis of single cells within tissue sections similar to flow cytometry. The principle of the method and the algorithms used have been described in detail elsewhere.^27^ In brief, the DAPI channel is the master marker for the identification of all events. This mask is then used in a corrected form to measure staining intensity in the other channels. Cut-off values for FITC and RRX channels were defined according to isotype controls, coexpressions were depicted in scattergrams of normalised grey values, and numbers of single positive and double positive cells were calculated as cells per mm^2^. To discriminate PCs and SMCs, parameters were chosen in order to identify αSMA positive cells within the basal lamina and outside.

### Statistical analysis

GraphPad Prism software V.6.0 was used for statistical analyses. Since the majority of our data did not pass the D'Agostino and Pearson omnibus normality test, two groups were compared by the non-parametrical Mann-Whitney U test after testing the global null hypothesis by the Kruskal-Wallis test. Local p values were adjusted by the global p value to preserve the family-wise error rate.^28^ p Values ≤0.05 were considered statistically significant.

## Results

### Primary outcome

**Figure 1:**
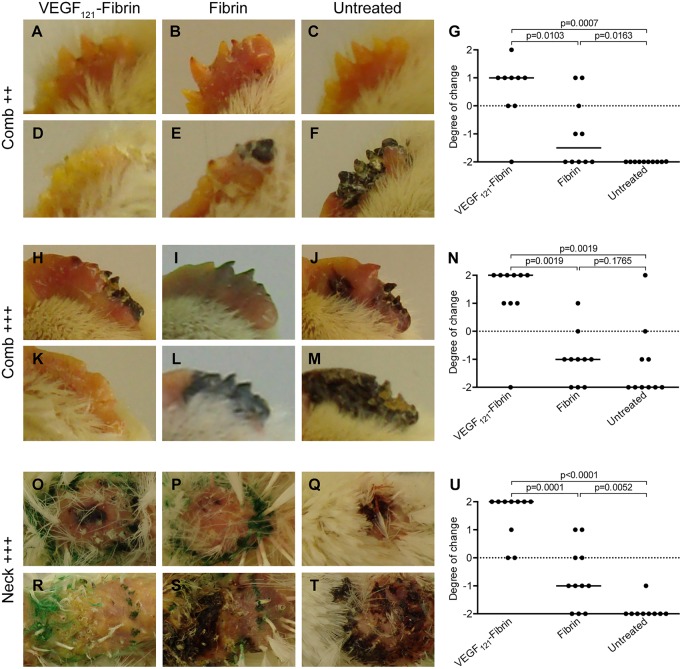
Clinical outcome 7 days after treatment. Representative examples of comb and neck lesions photographed before (A–C, H–J, O–Q), and after 1 week of treatment (D–F, K–M, R–T). Lesions at beginning of therapy: ++, erythema and oedema; +++, beginning necrosis. Results of clinical assessment by three blinded examiners of comb ++ (G), comb +++ (N), and neck +++ (U). Degree of change: 0=no change, +1=improvement, +2=strong improvement, −1=deterioration, −2=strong deterioration. p Values have been calculated using the Mann-Whitney U test adjusted by the Kruskal-Wallis test. Each dot represents a single lesion. Horizontal lines indicate the median. VEGF, vascular endothelial growth factor.

To evaluate whether VEGF_121_ modified fibrin heals and prevents ischaemic ulcerations, we locally treated early inflammatory comb lesions (C++), and late comb and neck skin ulcers (C+++, N+++) of UCD-206 chickens. After 1 week of treatment the clinical outcome was assessed (figure 1). Two animals treated with VEGF_121_-fibrin had lost the dressing and therefore were excluded from the study, that is, one with C++ lesion and one with N+++ lesion. From the comb lesions treated with VEGF_121_-fibrin in the early inflammatory C++ stage, six (66.7%) showed improvement, one (11.1%) deterioration and two (22.2%) halt of disease progression. Fibrin treated C++ lesions improved in two chickens (20.0%), worsened in seven (70.0%) and showed no change in one animal. All 10 untreated C++ lesions deteriorated. Nine comb lesions (90.0%) treated with VEGF_121_-fibrin in the late ischaemic C+++ stage showed improvement, one lesion deteriorated. In the fibrin treated group only one C+++ lesion improved slightly, eight (80.0%) worsened and one (10.0%) was unchanged. In the untreated C+++ group there was one (10.0%) spontaneous healing, one without change and eight (80.0%) with clear deterioration. Ulcerations of neck skin were clearly improved in eight animals (80.0%) after VEGF_121_-fibrin treatment, and showed no change in two chickens. Fibrin treatment improved only two N+++ lesions (18.2%), two (18.2%) were unaltered and seven (63.6%) progressed further. All 10 untreated lesions were strongly deteriorated. There was 100% agreement between the three blinded independent investigators in the semiquantitative clinical assessment in 60% of the cases; in 38% of the cases, two of the investigators showed 100% concordance, one differed by one degree up or down. The three investigators disagreed only in two cases on the severity of deterioration.

### Effect of VEGF_121_-fibrin on angiogenesis

**Figure 2:**
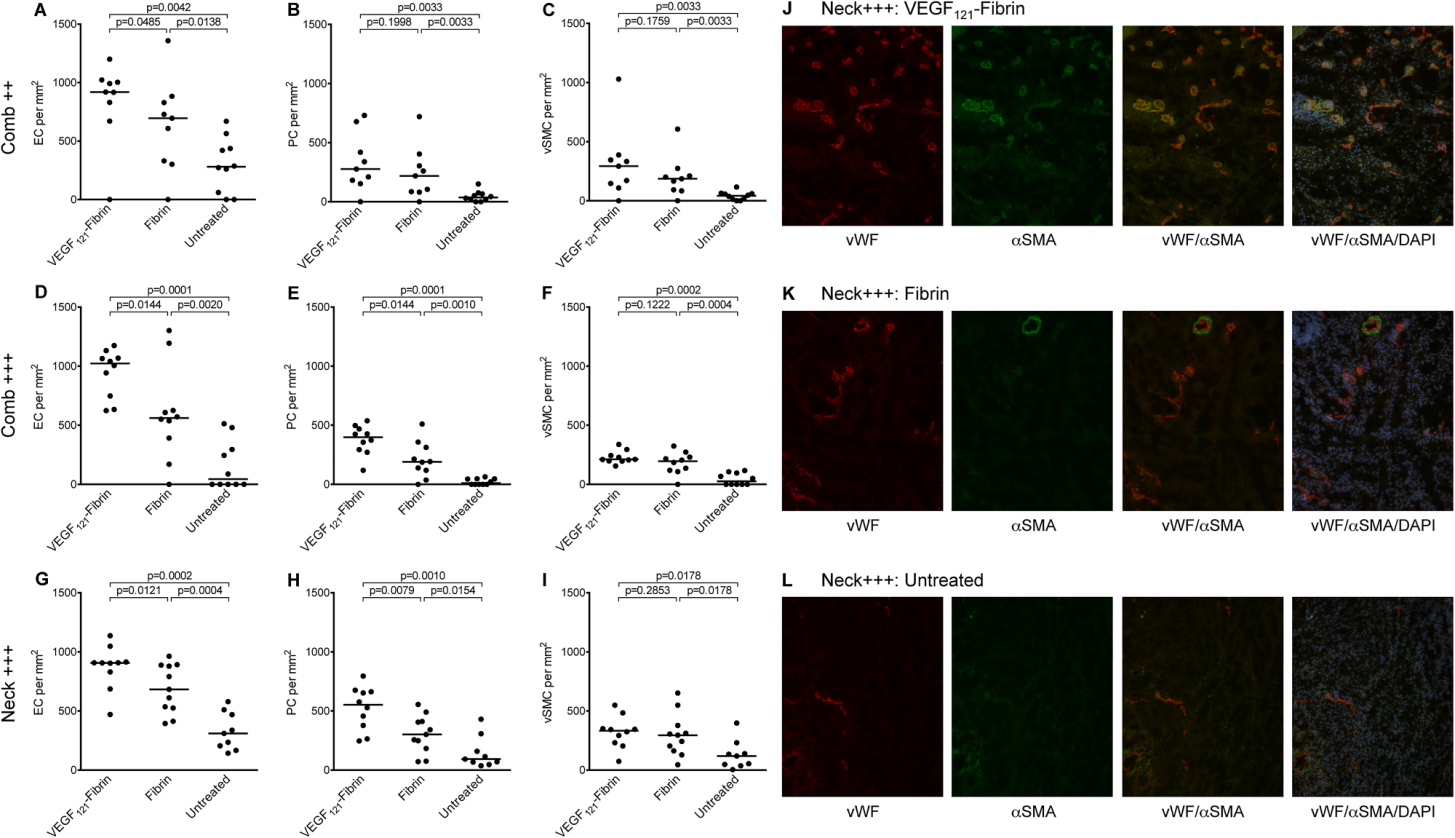
Effects of VEGF-therapy on angiogenesis**.** (A–I) display quantitative analyses of endothelial cells (ECs), pericytes (PCs) and vascular smooth muscle cells (vSMCs) after 1 week of treatment of early inflammatory comb lesions (C++; A–C), of comb ulcers (C+++; D–F), and neck ulcers (N+++, G–I). p Values have been calculated using the Mann-Whitney U test adjusted by the Kruskal-Wallis test. Each dot represents a single lesion. Horizontal bars indicate median values. Representative false colour immunofluorescence pictures of neck skin ulcers treated locally with VEGF_121_-fibrin (J), fibrin (K) or left untreated (L) depicting vWF staining, α smooth muscle actin (αSMA) staining, vWF/αSMA overlays and vWF/αSMA/DAPI overlays. Original magnification ×200. VEGF, vascular endothelial growth factor; vWF, von Willebrand factor.

To assess whether VEGF_121_-fibrin induces the growth of mature blood vessels, we quantitatively analysed frozen tissue sections stained by IIF with EC specific anti-vWF antibodies and anti-αSMA antibodies as a marker for mural cells. One of the fibrin treated C++ samples was lost due to accidental defrosting, and thus not included in the studies on angiogenesis. Microvascular density was significantly increased in VEGF_121_-fibrin treated early comb lesions, late comb lesions, and neck skin ulcers compared with fibrin treated and untreated lesions (figure 2A, D, G). The difference was also significant between VEGF_121_-fibrin and fibrin treated samples. Fibrin itself also increased the number of ECs significantly compared with untreated lesions. PC numbers were significantly elevated in all VEGF_121_-fibrin treated groups compared with untreated controls (figure 2B, E, H). Fibrin alone increased the number of PCs as well, but to a lesser extent. In late comb and neck ulcers, however, the effect of VEGF_121_-fibrin was significantly higher than of fibrin only. Significantly more vascular SMCs (vSMCs) were found in all VEGF_121_-fibrin treated lesions, and fibrin only groups compared with untreated controls (figure 2C, F, I). Increase of mural cell numbers after treatment with VEGF_121_-fibrin was confirmed by desmin staining (see online supplementary figure S1).

**Figure 3:**
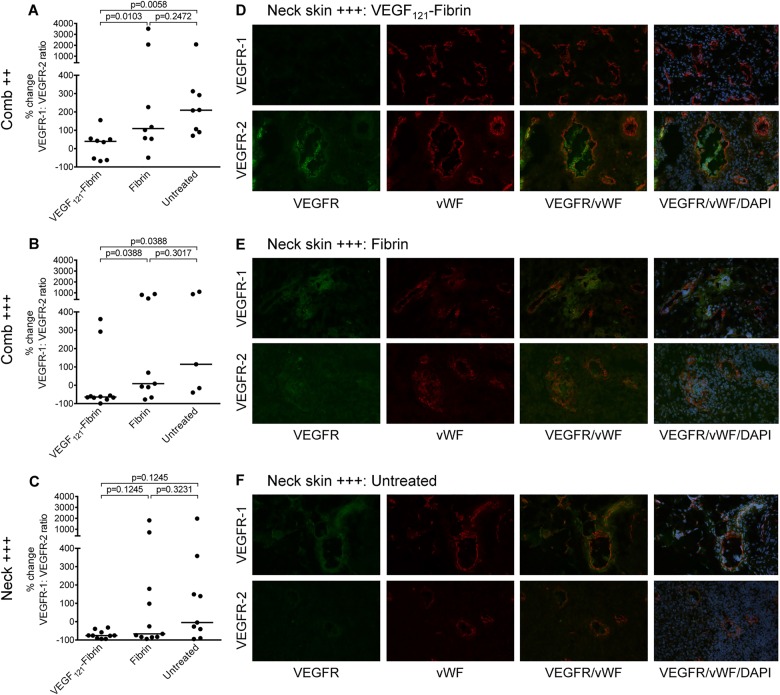
Endothelial expression of VEGFR-1 and VEGFR-2. Changes in the ratio of VEGFR-1 to VEGFR-2 expressing endothelial cells after 1 week of treatment of early inflammatory comb lesions (C++; A), of comb ulcers (C+++; B) and neck ulcers (N+++; C). Data expressed as change of the VEGFR-1:VEGFR-2 ratio in % from healthy control chickens, where the mean value of healthy chickens was defined as 0% change. Note that some deteriorated lesions showed no VEGFR-1 and VEGFR-2 expression, so that it was not possible to calculate the VEGFR-1:VEGFR-2 ratio. p Values have been calculated using the Mann-Whitney U test adjusted by the Kruskal-Wallis test. Each dot represents a single lesion. Horizontal bars indicate median values. Representative false colour immunofluorescence pictures of neck skin ulcers treated locally with VEGF_121_-fibrin (D), fibrin (E) or left untreated (F) showing single channel and overlays; upper panels: VEGFR-1, lower panels: VEGFR-2. Original magnification ×200. VEGF, vascular endothelial growth factor; vWF, von Willebrand factor.

**Figure 4:**
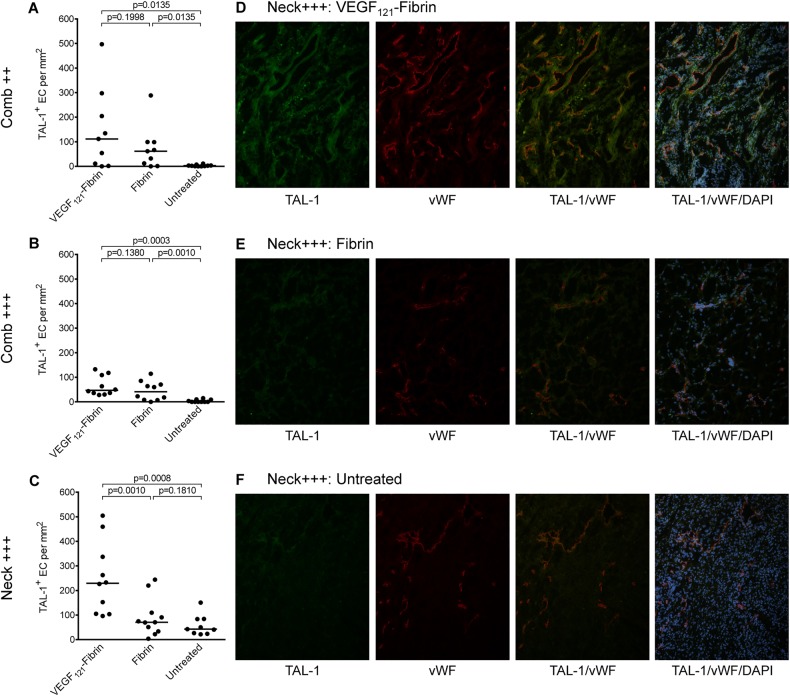
Endothelial expression of the VEGFR-2 regulator TAL-1. TAL-1 expression in endothelial cells (ECs) was quantified on immunofluorescence TAL-1/vWF double stained frozen tissue sections 1 week after treatment of early inflammatory comb lesions (C++; A), of comb ulcers (C+++; B) and neck ulcers (N+++; C). p Values have been calculated using the Mann-Whitney U test adjusted by the Kruskal-Wallis test. Each dot represents a single lesion. Horizontal bars indicate median values. Representative false colour immunofluorescence pictures of neck skin ulcers treated locally with VEGF_121_-fibrin (D), fibrin (E) or left untreated (F) showing single channel and overlays. Original magnification ×200. VEGF, vascular endothelial growth factor; vWF, von Willebrand factor.

To study the regulatory effects of VEGF_121_-fibrin on the expression of VEGF receptors VEGFR-1 and VEGFR-2, we determined the numbers of VEGFR-1/vWF double stained cells and the numbers of VEGFR-2/vWF double stained cells using TissueQuest, and calculated first the ratio of VEGFR-1 to VEGFR-2 expressing ECs. We then calculated the change of this ratio (x) in relation to healthy controls with the equation 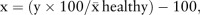 where y is the VEGFR-1:VEGFR-2 ratio of individual samples. Whereas in the majority of untreated lesions the VEGFR-1:VEGFR-2 ratio was increased in relation to healthy controls, the change of the VEGFR-1:VEGFR-2 ratio was significantly lower after VEGF_121_-fibrin treatment compared with untreated combs, and fibrin treated comb lesions (figure 3A, B). Neck skin lesions also showed a clear change of VEGFR-1:VEGFR-2 ratio after treatment with VEGF_121_-fibrin compared with fibrin and untreated controls. However the significance between groups was lost after Kruskal-Wallis adjustment (VEGF_121_-fibrin: median=−75.2 (IQR=−90.9 to −53.0), fibrin: −66.5 (−83.1 to 179.1); figure 3C–F). The reduction of the VEGFR-1:VEGFR-2 ratio was in part due to increased VEGFR-2 expression. To confirm this finding we also analysed the expression of TAL-1/SCL, a positive regulator of VEGFR-2 (figure 4). Compared with untreated controls VEGF_121_-fibrin increased the number of TAL-1 expressing ECs in early comb lesions, late comb lesions and neck ulcers. In the latter, the difference between VEGF_121_-fibrin and fibrin treatment was also statistically significant, whereas in comb lesions TAL-1 expressing ECs were elevated by fibrin compared with untreated controls.

**Figure 5:**
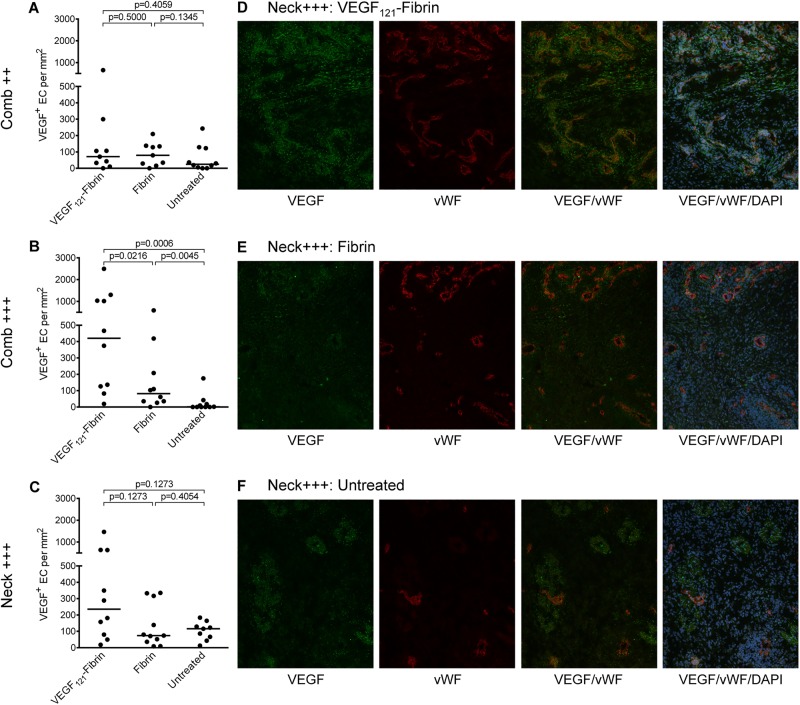
Endogenous VEGF expression in endothelial cells (ECs). Endogenous VEGF expression by ECs was quantified on immunofluorescence VEGF/vWF double stained frozen tissue sections after 1 week of treatment of early inflammatory comb lesions (C++; A), of comb ulcers (C+++; B) and neck ulcers (N+++; C). p Values have been calculated using the Mann-Whitney U test adjusted by the Kruskal-Wallis test. Each dot represents a single lesion. Horizontal bars indicate median values. Representative false colour immunofluorescence pictures of neck skin ulcers treated locally with VEGF_121_-fibrin (D), fibrin (E) or left untreated (F) showing single channel and overlays. Original magnification ×200. VEGF, vascular endothelial growth factor; vWF, von Willebrand factor.

Furthermore, we wanted to know if the exogenous VEGF_121_-fibrin has an influence on VEGF production by ECs. The number of VEGF expressing ECs was increased after VEGF_121_-fibrin treatment of comb and neck ulcers compared with fibrin and untreated controls. These differences were clearly significant in C+++, but not significant after Kruskal-Wallis adjustment in neck lesions (VEGF_121_-fibrin: 235.4 (72.9–636.7), fibrin: 74.1 (36.0–316.8), untreated control: 116.4 (54.9–146.7); figure 5).

## Discussion

The novelty and attractiveness of the current approach lies in the bioavailability of VEGF on cellular demand. This specifically addresses an unmet need in SSc where we have the situation of insufficiently increased VEGF levels. Moreover, some of the overexpressed VEGF might be antiangiogenic.^29^ Both aspects are addressed by the fibrin-bound VEGF gel: it provides locally in the wound bed sufficient amounts of VEGF and at the same time, the release of VEGF is on demand of the cells and the local release is stopped after it is no longer required. Our study in UCD-206 chickens convincingly showed the clinical efficacy of the topical VEGF_121_-fibrin therapy. In most cases of early treatment, that is, in combs with oedema but no ulceration, VEGF_121_-fibrin prevented the development of ischaemic ulcers. In total, only two animals (6.9%) did not respond to VEGF_121_-fibrin at all, and showed the same disease progression as untreated animals. Spontaneous healing was observed in only one untreated chicken with comb ulcer. Fibrin gel alone had a moderate improving effect on five lesions (16.1%). Overall, 79.3% of the VEGF_121_-fibrin treated lesions showed clear clinical improvement, whereas 71.0% of fibrin treated controls and 93.1% of untreated lesions had deteriorated. It should be noted here that, although closed bred, UCD-206 chickens are not an inbred line, and thus, like humans do not react uniformly.

VEGF_121_-fibrin had effectively promoted angiogenesis, leading to increased microvascular density. This is also reflected by significantly more ECs in comb and neck lesions treated with VEGF_121_-fibrin compared with fibrin or untreated controls. Nascent blood vessels initially consist only of ECs and have to be stabilised by mural cells.^30^ Mural cells of capillaries are referred to as PCs, those of arteries, arterioles, and veins as vSMCs. They both express αSMA, but can be distinguished by their localisation.^31^ We have set the parameters of the image analysis software TissueQuest in order to discriminate vSMCs, which are separated from ECs by the basement membrane or in larger arteries by the intima, and PCs, which share the basement membrane with the ECs and are in direct contact with these. Whereas only few mural cells were detected in untreated lesions, all the blood vessels of VEGF_121_-fibrin treated ulcers looked ensheathed by PCs or vSMCs suggesting the formation of mature, stable vessels. Desmin staining corroborated these findings.

The fibrin glue Tisseel, which we used to form the fibrin matrix, stimulated angiogenesis itself. Fibrin is the major component of blood clots, and serves as a provisional matrix during wound healing. Fibrin supports every stage of angiogenesis, that is, migration of ECs, tube formation and vessel maturation. In a rabbit model of hind limb ischaemia it has been found to promote angiogenesis even without the addition of any other proangiogenic factor.^32^ Thrombin, another component of Tisseel, as well as FXIII are also known to mediate angiogenesis.^33^ ^34^ FXIII was used to covalently bind VEGF_121_ to fibrin, but was also added to the placebo control. In vitro and in vivo studies have revealed that FXIII promotes migration and proliferation of ECs, inhibits apoptosis, and downregulates the antiangiogenic factor TSP-1 via VEGFR-2 activation.^34^ ^35^ Thus, all three components might contribute to the therapeutic effect of VEGF_121_-fibrin. However, VEGF_121_-fibrin showed significantly greater efficacy than the fibrin sealant with FXIII, especially in the treatment of late ischaemic ulcers of comb and neck skin.

Therapy with VEGF_121_-fibrin affected the expression of VEGFR-1 and VEGFR-2. Most of the VEGF signalling described to date is primarily mediated via VEGFR-2, that is, survival, migration, proliferation and vascular tube formation.^36^ In developmental angiogenesis VEGFR-1 acts as decoy receptor negatively regulating VEGFR-2 signalling.^37^ The role of VEGFR-1 in postnatal angiogenesis is less clear, and seems to be context dependent. However, several lines of evidence suggest an association of increased VEGFR-1 expression with impaired angiogenesis. VEGFR-1 levels are increased in chronic non-healing wounds, whereas in normal healing wounds granulation tissue formation is positively correlated with a decline in VEGFR-1.^38^ Elevated VEGFR-1 levels have been reported to promote endothelial injury in children with lupus nephritis,^39^ and to inhibit endothelial repair in PR3-ANCA associated vasculitis.^40^ Upregulation of VEGFR-1 and VEGFR-2 was demonstrated in SSc skin.^14^ ^41^ However, some of these semiquantitative results are contradictory. Whereas one study described a more pronounced VEGFR-2 expression, the other found more VEGFR-1. In bone marrow, diminished angiogenesis was associated with decreased VEGFR-2 expression and high VEGF levels.^42^ It is possible that differences between patients and the small sample numbers account for diverging results, but in general, they indicate activation of the VEGF/VEGFR system and imbalanced expression of VEGF and its receptors. VEGF action is regulated by the availability of its receptors. Therefore, it seems very likely that the ratio of VEGFR-1:VEGFR-2 rather than the absolute numbers determines the angiogenic status in the tissue. In lesional UCD-206 comb and neck skin the balance between the two receptors is shifted towards VEGFR-1. On exposure to VEGF_121_-fibrin the VEGFR-1:VEGFR-2 ratio was normalised due to differential regulation of VEGFR-1 and VEGFR-2 expression with a relative increase in endothelial VEGFR-2. Upregulation of VEGFR-2 expression concurred with increased TAL-1 expression. TAL-1 is a basic-helix-loop-helix transcription factor known to be essential for haematopoietic development. It is also required during vascular development and angiogenesis, and has been identified as a positive regulator of VEGFR-2.^43^ Moreover, endogenous VEGF expression was induced by modified VEGF_121_ in comb and neck skin ulcers. As autocrine VEGF signalling is required for endothelial survival,^44^ this might indicate the induction of lasting vascularisation.

All these results support the notion that cell-demanded release of fibrin-bound VEGF is capable of translating a supraphysiological dose into a physiological tiny dose, of sustaining this dose long enough to permit vessels to mature into stable vessels, and of stopping it if no longer needed. Otherwise chronic exposure would have harmful effects again. Furthermore, this study indicates that even a singular local administration of VEGF_121_-fibrin can achieve sufficient revascularisation to improve existing ulcers or prevent the development of ulcers in our SSc animal model. For clinical applications, long-term effects and potential side effects of VEGF_121_ need particular attention.

## Supplementary Material

Web supplement

Web figures
