# Supplementary material for: Efficient therapy of ischaemic lesions with VEGF121-fibrin in an animal model of systemic sclerosis
Source: Ann Rheum Dis. 2015 Sep 11;75(7):1399–406. doi: 10.1136/annrheumdis-2015-207548 (PMC4766736; doi:10.1136/annrheumdis-2015-207548)
Supplement: Web supplement [file annrheumdis-2015-207548-s1.pdf]

Supplementary Materials and Methods

Immunofluorescence stainings

For lack of anti-chicken antibodies, chicken cross reactive antibodies directed against human vWF, αSMA, desmin, VEGF, VEGFR-1, TAL-1, and murine VEGFR-2 have been used, i.e. polyclonal rabbit anti-human vWF, monoclonal mouse anti-human desmin (both DakoCytomation, Glostrup, Denmark), fluorescein isothiocyanate (FITC) conjugated monoclonal mouse anti-αSMA (SIGMA, Saint Louis, Missouri, USA), monoclonal mouse anti-human VEGF, clone 26503.11 recognizing $VEGF_{165}$ and $VEGF_{121}$ (SIGMA, Saint Louis, Missouri, USA), monoclonal mouse anti-human VEGFR-1, mouse anti-mouse VEGFR-2, and mouse anti-human TAL-1 (all Santa Cruz Biotechnology, Inc., California, USA). Isotype controls were purchased from R&D Systems (Minneapolis, USA) and Southern Biotech (Alabama, USA). Rhodamine Red-X (RRX) conjugated AffiniPure F(ab')2 fragment donkey anti-rabbit IgG and FITC conjugated AffiniPure F(ab')2 fragment donkey anti-mouse IgG (Jackson ImmunoResearch Laboratories, Inc., USA) were used as secondary antibodies. All antibodies and conjugates were diluted to predetermined optimal concentrations in phosphate buffered saline (PBS, pH 7.2) with 1% IgG-free bovine serum albumin (BSA, Jackson ImmunoResearch Laboratories, Inc., USA). Frozen tissue sections were mounted on 3-aminopropyl-triethoxysilane (SIGMA, Saint Louis, Missouri, USA) coated slides, air dried for 30 min, and incubated with either anti-VEGF, anti-VEGFR-1, anti-VEGFR-2, anti-TAL-1, anti-desmin or the respective isotype control over night at 4°C, or anti-αSMA for 30 min at room temperature (RT°), washed 30 min in PBS, incubated for 30 min with FITC-conjugated secondary antibody at RT°, and washed in PBS for 30 min. All samples were double stained with rabbit-anti-vWF and RRX-conjugated secondary antibody, incubation and washing steps 30 min at RT°. Nuclei were stained with DAPI (250 ng/ml, SIGMA, Saint Louis, Missouri, USA) for 5 min at RT°, and slides were mounted with Mowiol (Calbiochem, Nottingham, UK).
