# Supplementary material for: Efficient therapy of ischaemic lesions with VEGF121-fibrin in an animal model of systemic sclerosis
Source: Ann Rheum Dis. 2015 Sep 11;75(7):1399–406. doi: 10.1136/annrheumdis-2015-207548 (PMC4766736; doi:10.1136/annrheumdis-2015-207548)
Supplement: Web figures [file annrheumdis-2015-207548-s2.pdf]

Supplementary Figures

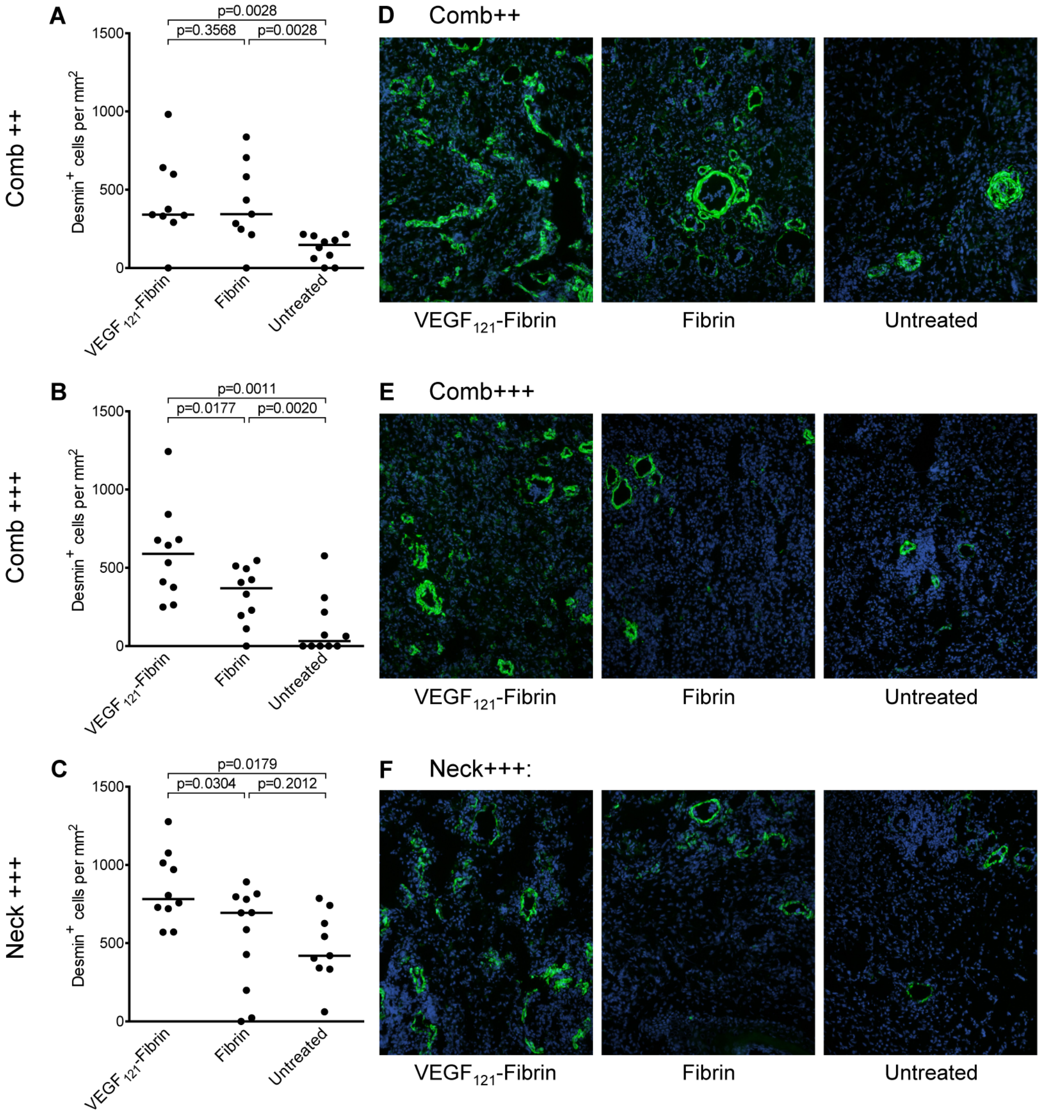

Figure S1 $VEGF_{121}$-fibrin treatment leads to growth of stable blood vessels. Desmin stained mural cells were quantified on immunofluorescence stained frozen tissue sections after one week of treatment of early inflammatory comb lesions (C++; A), of comb ulcers (C+++; B), and neck ulcers (N+++; C). *P* values have been calculated using the Mann-Whitney-U test adjusted by the Kruskal-Wallis test. Each dot represents a single lesion. Horizontal bars indicate median values. Representative false colour overlay pictures of desmin and DAPI stainings (D-F). Original magnification 200x.
